# Association of CHMP4B and Autophagy with Micronuclei: Implications for Cataract Formation

**DOI:** 10.1155/2014/974393

**Published:** 2014-03-11

**Authors:** Antonia P. Sagona, Ioannis P. Nezis, Harald Stenmark

**Affiliations:** ^1^Centre for Cancer Biomedicine, Faculty of Medicine, University of Oslo, Montebello, 0310 Oslo, Norway; ^2^Department of Biochemistry, Institute for Cancer Research, Oslo University Hospital, Montebello, 0310 Oslo, Norway; ^3^Division of Biomedical Cell Biology, Warwick Medical School, University of Warwick, Coventry CV4 7AL, UK; ^4^School of Life Sciences, University of Warwick, Coventry CV4 7AL, UK

## Abstract

Autophagy is a mechanism of cellular self-degradation that is very important for cellular homeostasis and differentiation. Components of the endosomal sorting complex required for transport (ESCRT) machinery are required for endosomal sorting and also for autophagy and the completion of cytokinesis. Here we show that the ESCRT-III subunit CHMP4B not only localizes to normal cytokinetic bridges but also to chromosome bridges and micronuclei, the latter surrounded by lysosomes and autophagosomes. Moreover, CHMP4B can be co-immunoprecipitated with chromatin. Interestingly, a CHMP4B mutation associated with autosomal dominant posterior polar cataract abolishes the ability of CHMP4B to localize to micronuclei. We propose that CHMP4B, through its association with chromatin, may participate in the autophagolysosomal degradation of micronuclei and other extranuclear chromatin. This may have implications for DNA degradation during lens cell differentiation, thus potentially protecting lens cells from cataract development.

## 1. Introduction

Autophagy is an evolutionarily conserved process where the cells degrade their own cellular material. It is involved in protein and organelle degradation and plays an essential role in cellular and whole-animal homeostasis and differentiation. There are various types of autophagy such as macroautophagy, microautophagy, and chaperone-mediated autophagy (for a comprehensive review see [1]). During autophagy there is sequestration of cellular material into double-membrane vesicles called autophagosomes. The autophagosomes fuse with endocytic vesicles to form the amphisomes, which contain both endocytic and autophagic cargo. The autophagosomes and/or amphisomes are subsequently fused with the lysosomes where the sequestered cargoes are degraded by lysosomal hydrolases. The products of degradation are transported back into the cytoplasm through lysosomal membrane permeases and can be reused by the cell [1]. Autophagy serves as a cellular response in nutrient starvation but is also responsible for the removal of aggregated proteins and damaged organelles and therefore plays an important role in the quality control of proteins and organelles. Dysfunctional autophagy is implicated in ageing, neurodegeneration, infections, tumorigenesis, heart disease, liver and lung disease, myopathies, and cataract formation [2] and it is therefore important to characterize this process at the molecular level.

The endosomal sorting complex required for transport (ESCRT) machinery is required for multivesicular body (MVB) biogenesis, budding of HIV-1 and other enveloped viruses, macroautophagy, and cytokinesis [3, 4]. The ESCRT machinery consists of four complexes: ESCRT-0, ESCRT-I, ESCRT-II, and ESCRT-III [3, 4]. ESCRT-III is specifically important for membrane scission events [5]. Based on electron microscopy studies, the ESCRT-III proteins CHMP4A and CHMP4B are able to assemble into filaments that curve and form circular arrays [6]. These membrane-associated ESCRT-III polymers can delineate and generate vesicles within the lumen of MVB and participate in the membrane scission process [6]. This ability of ESCRT-III to catalyze membrane scission applies to its role in other processes as well, such as cytokinesis and viral budding. The ESCRT-III component CHMP4B has been found to play a very important role for the final step of abscission during cytokinesis [7–9].

Completion of cytokinesis by abscission depends on the complete clearance of chromatin from the intercellular bridge and can be significantly delayed by lagging or bridged chromosomes [10]. Such defects occur in about 1% of dividing somatic cells and at higher incidence in transformed cells [11, 12]. Chromosome bridges and micronuclei often occur during genotoxic events and chromosomal instability [13]. Chromosome bridges originate during anaphase, either due to defective separation of sister chromatids or due to dicentric chromosomes which are formed because of misrepair of DNA breaks and telomere end fusions [13]. Micronuclei originate during anaphase from lagging acentric chromosome or chromatid fragments which result from unrepaired or misrepaired DNA breaks [13]. Whole chromosomes that fail to be included in the daughter nuclei at the completion of telophase during mitosis can also lead to micronuclei formation [13]. Importantly, micronuclei can also arise from chromosome bridges [14]. Chromosomes in these bridges are usually prone to break into multiple fragments and often these fragments form micronuclei at the end of mitosis [14]. However, it is unclear how this process is regulated and what molecules are involved.

Cataract is a genetic disorder of the crystalline lens which leads to visual impairment [15]. In the eye lens, epithelial cells of the anterior surface of the lens differentiate into fiber cells in a process accompanied by changes in cell shape, expression of crystallines, and degradation of cellular organelles and DNA, which ensure the transparency of the lens. Degradation of DNA of lens epithelial cells during their terminal differentiation into fibre cells is not associated with cell division. DNA degradation in the lens requires DNase II-like acid DNase (DLAD), and DLAD-deficient mice are incapable of degrading DNA during lens differentiation. Since undigested DNA accumulates in the fiber cells, the DLAD deficient mice form cataract [15]. Interestingly, the gene that encodes CHMP4B protein,* CHMP4B*, is found mutated in autosomal dominant cataracts [16]. However, the molecular details of CHMP4B function during lens fiber cell differentiation and its association with cataract formation are not yet clarified.

Here, we provide evidence that CHMP4B is associated with both chromosome bridges and micronuclei and that autophagosomes and lysosomes accumulate around CHMP4B-positive micronuclei. This suggests that CHMP4B could mediate autophagolysosomal degradation of extranuclear chromatin. We also show that a cataract-associated mutation in CHMP4B abolishes its recruitment to micronuclei. This raises the possibility that impaired autophagolysosomal degradation of extranuclear chromatin could explain cataract formation in the absence of proper CHMP4B function.

## 2. Materials and Methods

### 2.1. Cell Culture and Transfections

Media and reagents for cell culture were purchased from Gibco. HeLa, Hep2, MCF7, and U2OS cells were grown in Dulbecco's modified Eagle's medium (DMEM) containing 10% foetal calf serum (FCS), 5 U mL^−1^ penicillin, and 50 *μ*g mL^−1^ streptomycin. NIH3T3 cells were grown in Quantum 333 medium, containing 2% bovine serum and HLEB-3 cells were grown in Eagle's minimum essential medium (EMEM) containing 20% foetal bovine serum (FBS). Transfection of HeLa cells was performed as described previously [17].

### 2.2. Confocal Fluorescence Microscopy

Immunofluorescence microscopy was performed using HeLa, Hep2, MCF-7, U2OS, NIH3T3, and HLEB-3 cells as previously described [17]. Rabbit anti-human CHMP4B antibody was used in 1 : 1000 dilution and was synthesized as described before [17] and rabbit anti-human CHMP3 antibody synthesized in the same way was used in dilution 1 : 1000. Mouse anti-human Aurora B antibody, used in 1 : 200 dilution, mouse anti-human H2B antibody used in dilution 1 : 400, and mouse anti-human Lamin A antibody used in 1 : 200 dilution were purchased from Abcam. Mouse anti-human a-tubulin antibody used in dilution 1 : 1000 and mouse anti-human FLAG epitope M2 antibody used in dilution 1 : 150 were from Sigma-Aldrich, UK. Mouse anti-human Lamp1 antibody was from DSHB and used in dilution 1 : 200 and LC3 antibody was from Nanotools and used in dilution 1 : 200. The secondary antibodies used were Cy3-labelled goat anti-rabbit antibody in 1 : 500 dilution and Cy2-labelled goat anti-mouse antibody in 1 : 200 dilution and they were purchased from Jackson ImmunoResearch.

DNA was stained with Hoechst 33342 or DAPI at final concentration 1 *μ*g/mL. For the scoring of micronuclei and nucleoplasmic bridges the following criteria were adopted from Fenech et al. [18]: (1) the diameter of the MNi should be less than one-third of the main nucleus; (2) MNi should have similar staining as the main nucleus; (3) MNi should be separated from or marginally overlap with the main nucleus and located in the cytoplasm; (4) nucleoplasmic bridges were considered to be nuclear remnants localized inside the cytokinetic bridge, with similar staining characteristics to nuclei.

### 2.3. Co-Immunoprecipitation Analysis

Rabbit antibody against CHMP4B or rabbit IgG (control) was rotated at RT (room temperature) with Protein A agarose beads for 1 h. The beads were washed two times with PBS and two times with 0.2 M triethanolamine, pH 8.2. Crosslinking was performed by rotating the beads in 0.2 M triethanolamine containing 3 mg/mL dimethyl pimelimidate at 4°C overnight. For the quenching of the unreacted beads, they were rotated with 10 mM ethanolamine, pH 8.2, at 4°C for 30 min. Then the beads were washed three times with PBS and were used for immunoprecipitation.

HeLa cells were grown confluent in 10-cm culture dishes and lysed in ice-cold lysis buffer (20 mM HEPES pH 7.2, 2 mM MgCl2, 100 mM NaCl, 0.1 mM EDTA, 0.1% Triton X-100) containing inhibitors [(*N*-ethylmaleimide, mammalian protease inhibitor mixture, phosphatase inhibitor cocktail I and II (Sigma-Aldrich))].The lysates were placed on ice and centrifuged at 10,000 g, 4°C, and the supernatant was added to the Protein A-coupled magnetic beads (Dynal, Invitrogen) which had been precoupled with rabbit antibody against CHMP4B or rabbit IgG as a control, in PBS Tween 20. Antibody coupled magnetic beads and cell lysates were gently mixed for 1 h at 4°C. The beads were then washed with lysis buffer, eluted in 4 × sample buffer plus 1 mM DTT at 95°C for 5 min. The eluted proteins were subsequently subjected to SDS-PAGE and immunoblotting as described previously [17].

### 2.4. Plasmid Constructs

All the CHMP4B-FLAG constructs were kindly provided by Phyllis I. Hanson [16].

### 2.5. Quantification of the Colocalization of CHMP4B Wild Type and Mutant Constructs with Micronuclei

For this experiment, cells were transfected with FLAG-CHMP4B full length construct wild type or FLAG-CHMP4B full length construct which contained the mutation D129V found in patients with cataract and in both cases, among the transfected cells, those that exhibit colocalization of the transfected construct with micronuclei or chromosome bridges (which were stained with Hoechst or DAPI) were quantified. In total, 651 transfected cells with FLAG-CHMP4B full length construct from 5 different experiments and 570 transfected cells with FLAG-CHMP4B-D129V full length construct from 5 different experiments were quantified. For the cells transfected with wild type FLAG-CHMP4B full length construct, in 553 cells (84.9%) there was colocalization between FLAG-CHMP4B and micronuclei or chromosome bridges, whereas for the mutant, 247 transfected cells (43.33%) showed colocalization of CHMP4B cataract mutant construct with micronuclei or chromosome bridges. The frequency of micronucleation in HeLa cells, deriving from quantification of 4506 control (untransfected) cells from 3 different experiments, was found to be 6.3%.

### 2.6. Statistical Analysis

Values are given as means and SD in all figures. The *P* values are calculated based on *t*-test.

## 3. Results

### 3.1. CHMP4B Localizes to Various Types of Intercellular Bridges in Interconnected Cells

**Figure 1:**
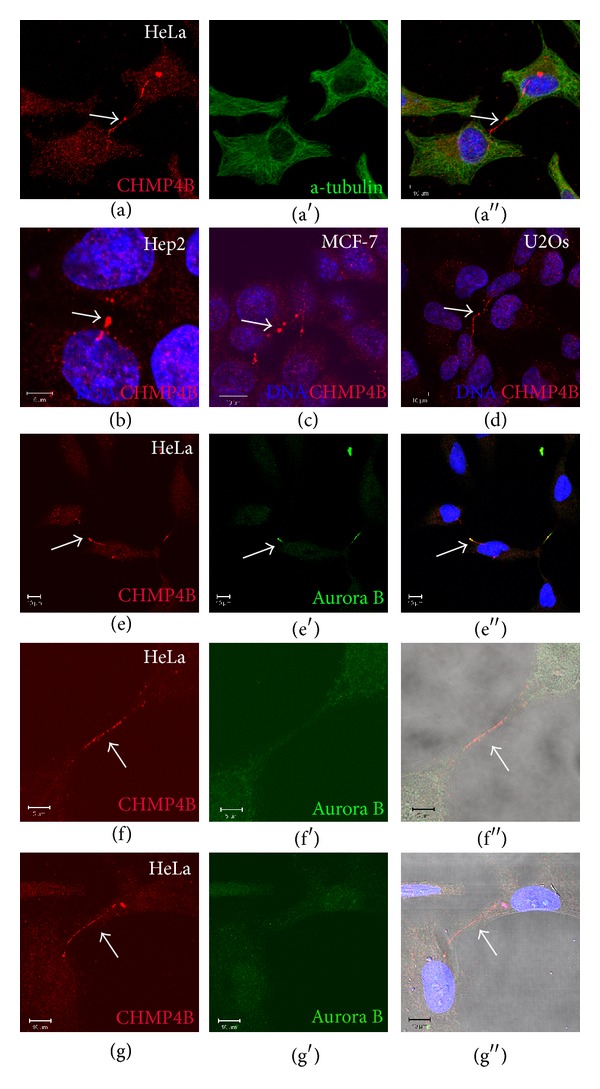
CHMP4B localizes to various types of intercellular bridges in interconnected cells. (a) Confocal micrographs of HeLa cells stained with CHMP4B, a-tubulin, and Hoechst. CHMP4B localizes to the intercellular bridge that links the cells (arrow). Scale bars: 10 *μ*m. (b)–(d) Confocal micrographs of Hep2, MCF-7 and U2Os cells, stained with CHMP4B and Hoechst. CHMP4B localizes to the bridge between the cells in all the above cell lines (arrows). Scale bars: 10 *μ*m. (e) Confocal micrographs of HeLa cells stained with CHMP4B, Aurora B and Hoechst. CHMP4B localizes to the intercellular bridge during cytokinesis (arrows). Scale bars: 10 *μ*m. (f)-(g) Confocal micrographs of HeLa cells stained with CHMP4B, Aurora B and Hoechst. CHMP4B localizes to thin (scale bars: 5 *μ*m) (f) and thick (scale bars: 10 *μ*m) (g) bridges (arrows) independently of Aurora B localization.

Previous studies have shown that CHMP4B localizes to the intercellular bridge adjacent to the midbody during cytokinesis in HeLa cells [7, 8, 17]. The availability of a sensitive and highly specific antibody against CHMP4B allowed us to perform a more detailed study of the localization of this protein by confocal microscopy. CHMP4B was found to localize to various types of intercellular bridges in interconnected HeLa cells. This occurred in a small proportion of cells (5.1 ± 2.3%) but was consistent in all the series of experiments performed (*n* = 25) (Figure 1(a)). This staining pattern was also observed in other cell types like Hep2, MCF-7, and U2Os and with similar frequency (3.2 ± 1.8% in Hep2 cells, 3.7 ± 1.9% in MCF-7 cells, and 4.2 ± 1.2% in U2Os cells) (Figures 1(b)–1(d)). Our immunofluorescence analysis showed that CHMP4B localizes to thin (Figures 1(a), 1(e), and 1(f)) or thick (Figure 1(g)) bridges and exhibits a long filamentous localization pattern which appears to interconnect the cells and to emanate from close proximity of the nucleus. Often, CHMP4B accumulates in large structures at the starting points of each bridge (Figures 1(a) and 1(g)). This staining pattern occurs independently of Aurora B staining suggesting that these intercellular bridges represent consequent events of incomplete or defective cytokinesis (Figures 1(e)–1(g)). In order to examine whether this staining pattern is exclusive for CHMP4B, we also tested the localization of other ESCRT-III components, such as CHMP3. We observed that CHMP3 also forms filamentous structures inside intercellular bridges (see Supplementary Figures 1(a) and 1(b) in Supplementary Material available online at http://dx.doi.org/10.1155/2014/974393). The above data show that ESCRT-III components CHMP4B and CHMP3, apart from their canonical staining pattern at the midbody during cytokinesis, exhibit an additional staining pattern in various types of intercellular bridges that connect dividing cells.

### 3.2. CHMP4B but Not CHMP3 Associates with DNA in the Chromosome Bridges and in Micronuclei

**Figure 2:**
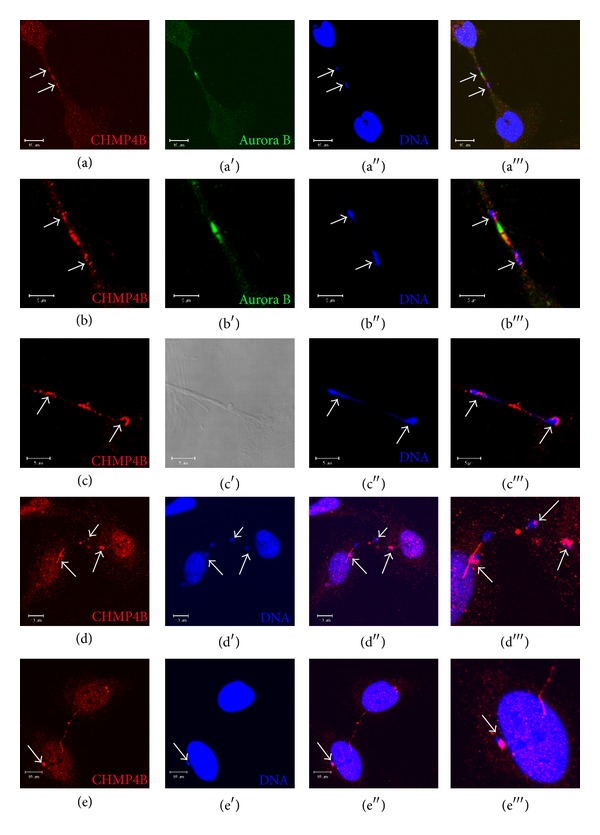
CHMP4B localizes to chromosome bridges and micronuclei. (a), (b) Confocal micrographs of HeLa cells stained with CHMP4B, Aurora B, and Hoechst. CHMP4B associates with chromosome bridges (arrows) (scale bars: 10 *μ*m) (a) as also presented in higher magnification (arrows) (scale bars: 10 *μ*m) (b). (c), (d) Confocal micrographs of HeLa cells, stained with CHMP4B and Hoechst. CHMP4B attaches to chromosome bridges (arrows) (scale bars: 5 *μ*m) (c) and colocalizes with the DNA present in the chromosome bridge (arrows) (scale bars: 10 *μ*m) (d). (e) Confocal micrographs of HeLa cells stained with CHMP4B and Hoechst. CHMP4B localizes to the micronucleus (arrow). Scale bars: 10 *μ*m.

During our immunofluorescence studies, we noticed that DNA is sometimes trapped inside intercellular bridges and forms chromosome bridges. In order to test the possible association of CHMP4B with the chromosomal bridges, we performed staining with CHMP4B and Hoechst or DAPI and we found that CHMP4B colocalizes strongly with DNA in the chromosome bridges (Figures 2(a)–2(d)). Interestingly, we observed that CHMP4B also colocalizes with micronuclei (Figures 2(d) and 2(e)). These structures often accumulate at the entry of the bridge suggesting that they can be derived from the chromosome bridge (Figure 2(d)). Importantly depletion of CHMP4A/B resulted in increased number of micronuclei (Supplementary Figure 2). Surprisingly, we observed that the ESCRT-III component CHMP3 did not associate with chromosome bridges or micronuclei (Supplementary Figures 1(c) and 1(d)). The above data show that the ESCRT-III component CHMP4B but not CHMP3 colocalizes with DNA found in chromosomal bridges and micronuclei.

### 3.3. CHMP4B Co-Immunoprecipitates with Histone Protein H2B and the Inner Nuclear Membrane Protein Lamin A

**Figure 3:**
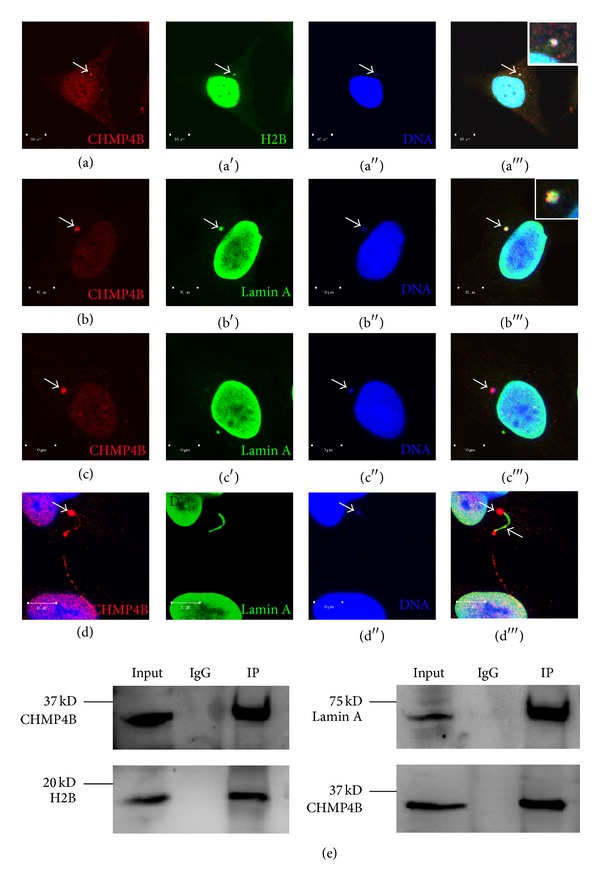
CHMP4B co-mmunoprecipitates with Histone 2B and Lamin A. (a) Confocal micrographs of HeLa cells stained with CHMP4B, Histone 2B, and DAPI. CHMP4B colocalizes with Histone 2B in the micronucleus (arrow). Magnification of the micronucleus is shown in the inset. Scale bars: 10 *μ*m. (b)–(d) Confocal micrographs of HeLa cells stained with CHMP4B, Lamin A, and DAPI. CHMP4B colocalizes in some cases with Lamin A in the micronucleus (arrow) (b) and in some cases does not colocalize with Lamin A (arrow) (c) and is localizing to the chromosome bridge stained by Lamin A (arrows) (d). Scale bars: 10 *μ*m. (e) HeLa cell lysates were subjected to immunoprecipitation (IP) with an antibody against CHMP4B. Immunoprecipitated proteins were detected by Western blotting, using anti-H2B, anti-Lamin A, and anti-CHMP4B antibodies.

In order to further investigate the association of CHMP4B with DNA, we performed double stainings with CHMP4B and various chromatin-associated proteins, such as Histone 2B and Lamin A. We observed as expected that CHMP4B colocalized with Histone 2B and Lamin A at the micronuclei (Figures 3(a) and 3(b)), even though in some cases it did not seem to colocalize with Lamin A (Figure 3(c)). CHMP4B also strongly accumulated at Lamin A positive chromosome bridges (Figure 3(d)).

To investigate whether there is a physical association between CHMP4B and chromatin, we performed immunoprecipitation experiments between CHMP4B and Histone 2B or Lamin A. These experiments clearly showed that CHMP4B associates physically with Histone 2B and Lamin A (Figure 3(e)). Taken together the above results show that CHMP4B strongly associates with chromatin.

### 3.4. Lamp1 and LC3 Localize Adjacent to Micronuclei

**Figure 4:**
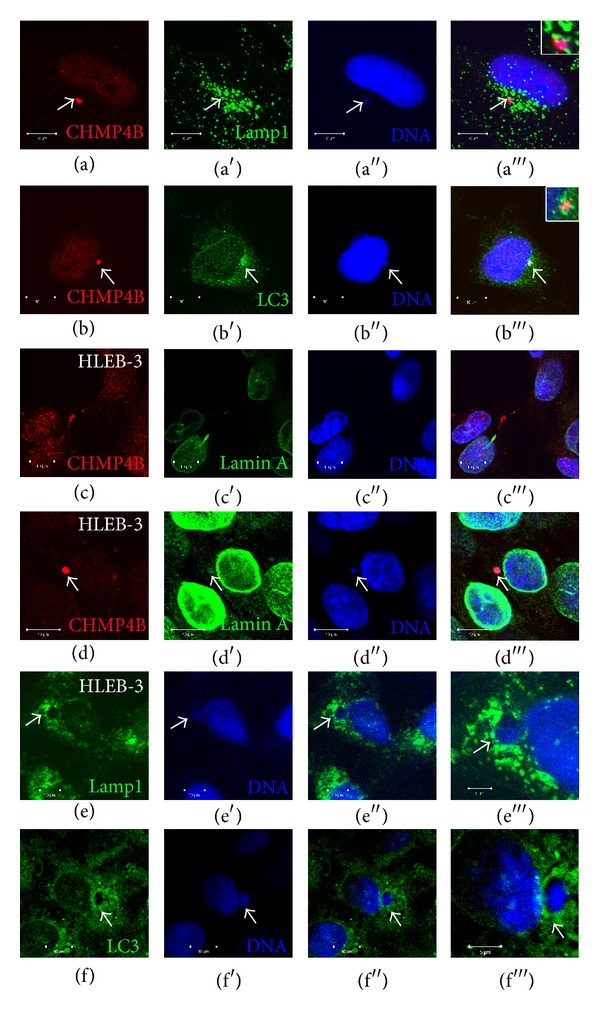
Lamp1 and LC3 localize adjacent to micronuclei in HeLa and HLEB-3 cells. (a) Confocal micrographs of HeLa cells stained with CHMP4B, Lamp1, and DAPI. Lamp1 positive lysosomes localize adjacent to CHMP4B positive micronucleus (arrow). Scale bars: 10 *μ*m. Magnification of the micronucleus stained with CHMP4B, Lamp1, and DAPI is shown in the inset. (b) Confocal micrographs of HeLa cells stained with CHMP4B, LC3, and DAPI. CHMP4B positive micronucleus is adjacent to LC3 (arrow). Scale bars: 10 *μ*m. Magnification of the micronuclei stained with CHMP4B, DAPI, and LC3 is shown in the inset. (c)-(d) Confocal micrographs of HLEB-3 cells stained with CHMP4B, Lamin A, and DAPI. CHMP4B is present on the intercellular bridge (c) and the micronucleus (d). Scale bars: 10 *μ*m. (e)-(f) Confocal micrographs of HLEB-3 cells stained with Lamp1, LC3, and DAPI. Lamp1 positive and LC3 positive structures localize adjacent to the micronuclei (arrows). Scale bars: 10 *μ*m.

CHMP4B was found mutated in autosomal dominant cataracts [16]. Cataract formation is associated with defective degradation of cellular organelles and DNA in the epithelial cells of the eye lens [15]. Since DNA degradation in the epithelial cells of eye lens was suggested to be mediated by the lysosomal machinery [19], we tested whether CHMP4B positive micronuclei are associated with lysosomal and autophagic markers. For this purpose, we co-stained HeLa cells with CHMP4B and the late endosomal/lysosomal marker Lamp1 (Figure 4(a)) as well as the autophagic marker LC3 (Figure 4(b)). We observed that both markers localize adjacent to CHMP4B positive micronuclei, suggesting that CHMP4B positive micronuclei may be degraded via the lysosomal machinery in HeLa cells (Figures 4(a) and 4(b)). Since this scenario fits with the proposed mechanism of DNA degradation during the differentiation of lens cells in the eye which protects them from the formation of cataract, we stained human epithelial lens cells HLE B-3 with CHMP4B to test its localization. We found that CHMP4B indeed localizes to micronuclei as well as to intercellular bridges in lens cells (Figures 4(c) and 4(d)). Additionally, the lysosomal/autophagic markers Lamp1 and LC3 were observed to localize adjacent to micronuclei in human epithelial lens cells HLE B-3, further supporting our findings in HeLa cells (Figures 4(e) and 4(f)).

### 3.5. A CHMP4B Mutation Found in Cataract Abolishes Its Localization to Chromosome Bridges and Micronuclei

**Figure 5:**
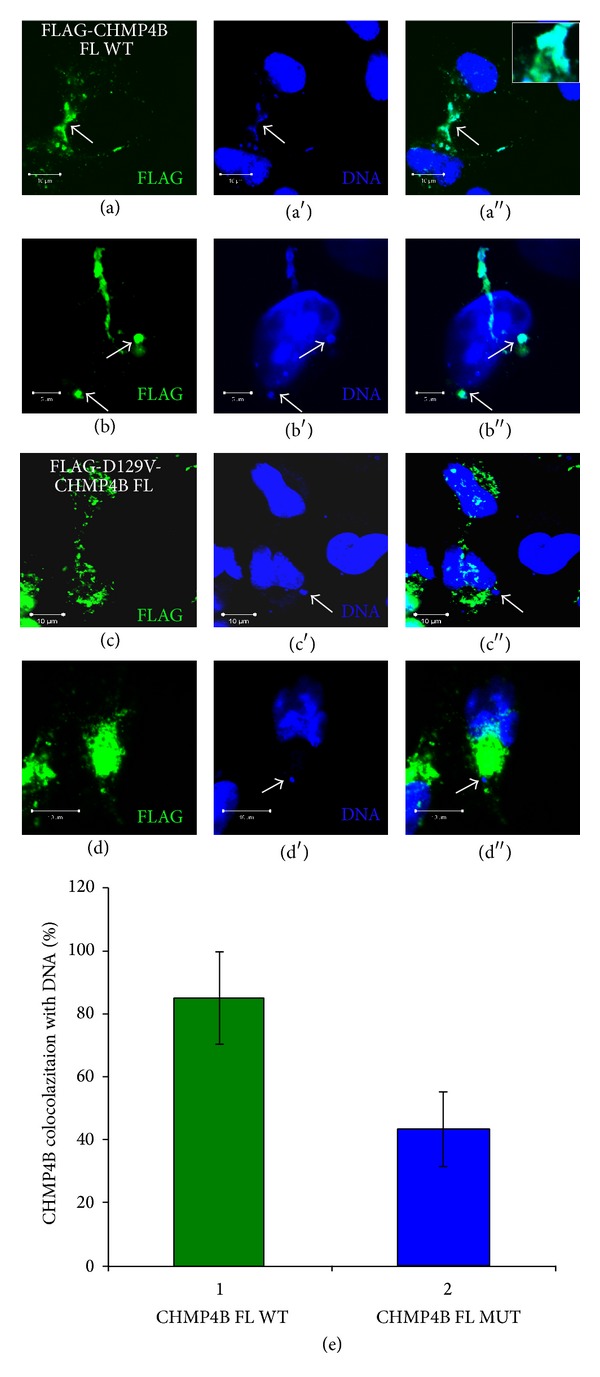
CHMP4B-mutant found in cataract shows reduced localization to chromosome bridges and micronuclei. (a)-(b) Confocal micrographs of HeLa cells transfected with FLAG-CHMP4B full length and stained with FLAG epitope M2 antibody and Hoechst. FLAG-CHMP4B full length associates strongly with chromosome bridges and micronuclei (a)-(b) (arrows). Scale bars: 10 *μ*m and 5 *μ*m. (c)-(d) Confocal micrographs of HeLa cells transfected with FLAG-D129V-CHMP4B full length and stained with FLAG epitope M2 antibody and Hoechst. FLAG-D129V-CHMP4B full length does not associate strongly with chromosome bridges and micronuclei (arrows). Scale bars: 10 *μ*m. (e) Graphic presentation of quantification of %CHMP4B colocalization with DNA in transfected cells with FLAG-CHMP4B full length wild type versus FLAG-CHMP4B-D129V full length mutant. Error bars show mean ± SD. FLAG-CHMP4B full length: 5 independent experiments, *n* = 651 cells. FLAG-CHMP4B-D129V full length: 5 independent experiments, *n* = 570 cells. *P* value for full length FLAG-constructs: 0.048. The *P* values were derived from comparing means by independent samples *t*-test (SPSS, v.16.0).

In order to further investigate the functional role of CHMP4B in cataract formation, we asked whether there is any difference in the ability of association with chromatin between the wild type and cataract mutant CHMP4B forms. For this purpose, we transfected HeLa cells with FLAG-CHMP4B full length construct and measured the colocalization of FLAG-CHMP4B with DNA chromosome bridges and micronuclei (Figures 5(a), 5(b), and 5(e)). We repeated the experiment with a FLAG-CHMP4B full length construct which contained the mutation D129V found in patients with cataract (Figures 5(c), 5(d), and 5(e)). We found that in average of five separate experiments, 84.9% of wild-type FLAG-CHMP4B transfected cells showed strong colocalization between the transfected protein and chromosome bridges or micronuclei, whereas with the mutant construct the percentage was significantly reduced to 43.3% (Figure 5(e)). These results suggest that CHMP4B mutant protein found in cataract has a defective association with chromatin.

## 4. Discussion

The ESCRT-III subunit CHMP4B plays a crucial role in the final abscission step during cytokinesis by participating in the formation of helical filaments that support the constriction of the intercellular bridge and the final abscission [7–9]. Here, we demonstrate a novel localization pattern of CHMP4B to chromosome bridges and micronuclei in various cell lines. This localization, together with our finding that lysosomes and autophagosomes accumulate around micronuclei, suggests the possibility that CHMP4B might mediate lysosomal degradation of extranuclear chromatin.

Micronuclei were shown to arise from chromosome bridges in cancer cell lines [14]. CHMP4B is the first non-nuclear protein to localize to both structures and thus connects failure of cytokinesis with micronuclei. The role of CHMP4B during this process will have to be addressed in detail in future studies, but it is interesting that ESCRT-III has previously been implicated in degradation of intracellular protein aggregates [20] suggesting a related mechanism for chromatin degradation.

Importantly, localization of CHMP4B to micronuclei was also observed in the HLEB-3 human epithelial lens cell line. The gene that encodes CHMP4B protein is found mutated in autosomal dominant cataract [16], a disease with unknown molecular mechanism, even though it is known that it is linked with unsuccessful degradation of cellular organelles and chromosomal DNA during lens cell differentiation from epithelial to fiber cells [15, 21]. Based on studies with mouse models, DNase II-like acid DNase (DLAD) has been shown to be responsible for the degradation of chromosomal DNA in the lens [19]. DLAD has been found to colocalize with the lysosomal marker Lamp1 [19], suggesting the possibility that degradation of DNA could occur via lysosomal degradation. Here we found that the lysosomal and autophagic markers Lamp1 and LC3 localize around CHMP4B positive micronuclei in HeLa and HLEB-3 cells, suggesting that micronuclei may be digested via lysosomal degradation.

Interestingly, the mutation in CHMP4B D129V found in cataract patients was shown to abolish its localization to micronuclei compared to the wild-type protein. This suggests that CHMP4B may have a role in mediating the degradation of micronuclei. We speculate that CHMP4B may facilitate the recruitment of lysosomes to micronuclei or the fusion of lysosomes with the micronuclear membrane. Since degradation of nuclei during lens cells differentiation has been associated with the lysosomal machinery [15, 19, 21, 22] we propose that CHMP4B participates in the lysosomal degradation of chromosomal DNA during lens cell differentiation, thus protecting from the formation of cataract. Organelle degradation during lens differentiation occurs independently of the canonical autophagy machinery, since it has been found to occur normally in ATG5 and PIK3C3/VPS34 deficient mice [23, 24]. In our experiments we have observed that autophagosomes localize adjacent to micronuclei. Autophagy may facilitate the degradation of small parts of the micronuclei. This observation is in agreement with the results reported by Nakahara et al. who showed that expression of the autophagy-related* atg3* and* atg4b* genes was significantly upregulated during fiber lens cells differentiation in mice [19]. Furthermore, it was recently shown that human lens expresses the full complement of genes required to carry out autophagy [25] and that these genes are expressed in both adult human lens epithelial cells and differentiating fiber cells. Additionally, ATG5-independent and PIK3C3/VPS34-independent autophagy have been reported to function in the autophagic elimination of organelles during erythrocyte differentiation and neuronal development [26, 27] raising the possibility that alternative non-canonical autophagy may participate also during lens differentiation. It has been also shown that mutations in the autophagy gene FYCO1 (FYVE and coiled coil domain containing 1) cause autosomal recessive congenital human cataract [28] and that autophagy and mitophagy have a role in ocular lens organelle degradation [28, 29]. Finally Rello-Varona and colleagues recently reported that micronuclei can be subjected to autophagic degradation [30]. These reports together with our present data suggest that autophagy is important for human lens cells differentiation.

Defects in cytokinesis may also cause cataract. There are reports showing that cell cycle arrest by kynurenine and expression of phosphorylation-compromised vimentin may play a role in cataract formation [31, 32]. Thus, given the role of CHMP4B in cytokinesis, we cannot exclude the possibility that defective CHMP4B function in lens cells might cause cataract due to cytokinesis failure.

In conclusion, in the present study we demonstrate that CHMP4B is a novel common component of chromosome bridges and micronuclei. We propose that CHMP4B may participate in the autophagolysosomal degradation of micronuclei, and this may have implications in DNA degradation during lens cell differentiation and cataract formation. Future studies will hopefully shed more light on the molecular details of these processes.

## Supplementary Material

Supplementary Figure 1: CHMP3 localizes at the midbody and intercellular bridge but does not colocalize with micronuclei.
Supplementary Figure 2: Depletion of CHMP4A/B results in increased number of micronuclei.

## References

[1] Yang Z, Klionsky DJ (2010). Eaten alive: a history of macroautophagy. *Nature Cell Biology*.

[2] Rubinsztein DC, Mariño G, Kroemer G (2011). Autophagy and aging. *Cell*.

[3] Raiborg C, Stenmark H (2009). The ESCRT machinery in endosomal sorting of ubiquitylated membrane proteins. *Nature*.

[4] Hurley JH, Hanson PI (2010). Membrane budding and scission by the ESCRT machinery: it’s all in the neck. *Nature Reviews Molecular Cell Biology*.

[5] Adell MAY, Teis D (2011). Assembly and disassembly of the ESCRT-III membrane scission complex. *FEBS Letters*.

[6] Hanson PI, Roth R, Lin Y, Heuser JE (2008). Plasma membrane deformation by circular arrays of ESCRT-III protein filaments. *Journal of Cell Biology*.

[7] Guizetti J, Schermelleh L, Mäntler J (2011). Cortical constriction during abscission involves helices of ESCRT-III-dependent filaments. *Science*.

[8] Elia N, Sougrat R, Spurlin TA, Hurley JH, Lippincott-Schwartz J (2011). Dynamics of endosomal sorting complex required for transport (ESCRT) machinery during cytokinesis and its role in abscission. *Proceedings of the National Academy of Sciences of the United States of America*.

[9] Neto H, Gould GW (2011). The regulation of abscission by multi-protein complexes. *Journal of Cell Science*.

[10] Steigemann P, Wurzenberger C, Schmitz MHA (2009). Aurora B-mediated abscission checkpoint protects against tetraploidization. *Cell*.

[11] Cimini D, Mattiuzzo M, Torosantucci L, Degrassi F (2003). Histone hyperacetylation in mitosis prevents sister chromatid separation and produces chromosome segregation defects. *Molecular Biology of the Cell*.

[12] Gisselsson D, Pettersson L, Höglund M (2000). Chromosomal breakage-fusion-bridge events cause genetic intratumor heterogeneity. *Proceedings of the National Academy of Sciences of the United States of America*.

[13] Fenech M, Kirsch-Volders M, Natarajan AT (2011). Molecular mechanisms of micronucleus, nucleoplasmic bridge and nuclear bud formation in mammalian and human cells. *Mutagenesis*.

[14] Hoffelder DR, Luo L, Burke NA, Watkins SC, Gollin SM, Saunders WS (2004). Resolution of anaphase bridges in cancer cells. *Chromosoma*.

[15] Nishimoto S, Kawane K, Watanabe-Fukunaga R (2003). Nuclear cataract caused by a lack of DNA degradation in the mouse eye lens. *Nature*.

[16] Shiels A, Bennett TM, Knopf HLS (2007). CHMP4B, a novel gene for autosomal dominant cataracts linked to chromosome 20q. *American Journal of Human Genetics*.

[17] Sagona AP, Nezis IP, Pedersen NM (2010). PtdIns(3)P controls cytokinesis through KIF13A-mediated recruitment of FYVE-CENT to the midbody. *Nature Cell Biology*.

[18] Fenech M, Chang WP, Kirsch-Volders M, Holland N, Bonassi S, Zeiger E (2003). HUMN project: detailed description of the scoring criteria for the cytokinesis-block micronucleus assay using isolated human lymphocyte cultures. *Mutation Research*.

[19] Nakahara M, Nagasaka A, Koike M (2007). Degradation of nuclear DNA by DNase II-like acid DNase in cortical fiber cells of mouse eye lens. *FEBS Journal*.

[20] Filimonenko M, Stuffers S, Raiborg C (2007). Functional multivesicular bodies are required for autophagic clearance of protein aggregates associated with neurodegenerative disease. *Journal of Cell Biology*.

[21] Nagata S, Kawane K (2011). Autoinflammation by endogenous DNA. *Advances in Immunology*.

[22] Vrensen GFJM, Graw J, De Wolf A (1991). Nuclear breakdown during terminal differentiation of primary lens fibres in mice: a transmission electron microscopic study. *Experimental Eye Research*.

[23] Matsui M, Yamamoto A, Kuma A, Ohsumi Y, Mizushima N (2006). Organelle degradation during the lens and erythroid differentiation is independent of autophagy. *Biochemical and Biophysical Research Communications*.

[24] Morishita H, Eguchi S, Kimura H (2013). Deletion of autophagy-related 5 (Atg5) and Pik3c3 genes in the lens causes cataract independent of programmed organelle degradation. *The Journal of Biological Chemistry*.

[25] Brennan LA, Kantorow WL, Chauss D (2012). Spatial expression patterns of autophagy genes in the eye lens and induction of autophagy in lens cells. *Molecular Vision*.

[26] Nishida Y, Arakawa S, Fujitani K (2009). Discovery of Atg5/Atg7-independent alternative macroautophagy. *Nature*.

[27] Zhou X, Wang F (2010). Effects of neuronal PIK3C3/Vps34 deletion on autophagy and beyond. *Autophagy*.

[28] Chen J, Ma Z, Jiao X (2011). Mutations in FYCO1 cause autosomal-recessive congenital cataracts. *American Journal of Human Genetics*.

[29] Costello MJ, Brennan LA, Basu S (2013). Autophagy and mitophagy participate in ocular lens organelle degradation. *Experimental Eye Research*.

[30] Rello-Varona S, Lissa D, Shen S (2012). Autophagic removal of micronuclei. *Cell Cycle*.

[31] Mailankot M, Smith D, Howell S (2008). Cell cycle arrest by kynurenine in lens epithelial cells. *Investigative Ophthalmology and Visual Science*.

[32] Matsuyama M, Tanaka H, Inoko A (2013). Defect of mitotic vimentin phosphorylation causes microophthalmia and cataract via aneuploidy and senescence in lens epithelial cells. *The Journal of Biological Chemistry*.

