# Supplementary material for: Association of CHMP4B and Autophagy with Micronuclei: Implications for Cataract Formation
Source: Biomed Res Int. 2014 Mar 11;2014:974393. doi: 10.1155/2014/974393 (PMC3967805; doi:10.1155/2014/974393)
Supplement: Supplementary file 1 — Supplementary Figure 1: CHMP3 localizes at the midbody and intercellular bridge but does not colocalize with micronuclei. Supplementary Figure 2: Depletion of CHMP4A/B results in increased number of micronuclei. [file 974393.f1.zip › Description.docx]

**Supplementary Figure 1**

**CHMP3 localizes at the midbody and intercellular bridge but does not colocalize with micronuclei.**

(**A**)-(**D**) Confocal micrographs of HeLa cells stained with Hoechst and with antibodies against CHMP3. CHMP3 colocalizes with Aurora B to the midbody (arrow) (A) and localizes to the bridge between the cells (arrow) (B) but does not seem to associate with micronuclei (arrows) (C),(D).

**Supplementary Figure 2**

**Depletion of CHMP4A/B results in increased number of micronuclei**

Western blot showing the down regulation of CHMP4A and CHMP4B after RNAi knock down. **(B)** Three different experiments with 8 coverslips (4 control and 4 KD) for each experiment 4506 control and 3421 depleted cells quantified manually. 6,36% of total numbers of micronuclei where counted in control cells and 13,06% in CHMP4A/CHMP4B depleted cells. 6,3% of cells were found to contain micronuclei in control cells and 12,16% of cells were found to contain micronuclei in CHMP4A/CHMP4B depleted cells. **(C)** Representative confocal micrographs showing the increase of micronuclei (arrows) in CHMP4A/CHMP4B depleted cells
