# Supplementary figures and images for: Association of CHMP4B and Autophagy with Micronuclei: Implications for Cataract Formation
Source: Biomed Res Int. 2014 Mar 11;2014:974393. doi: 10.1155/2014/974393 (PMC3967805; doi:10.1155/2014/974393)

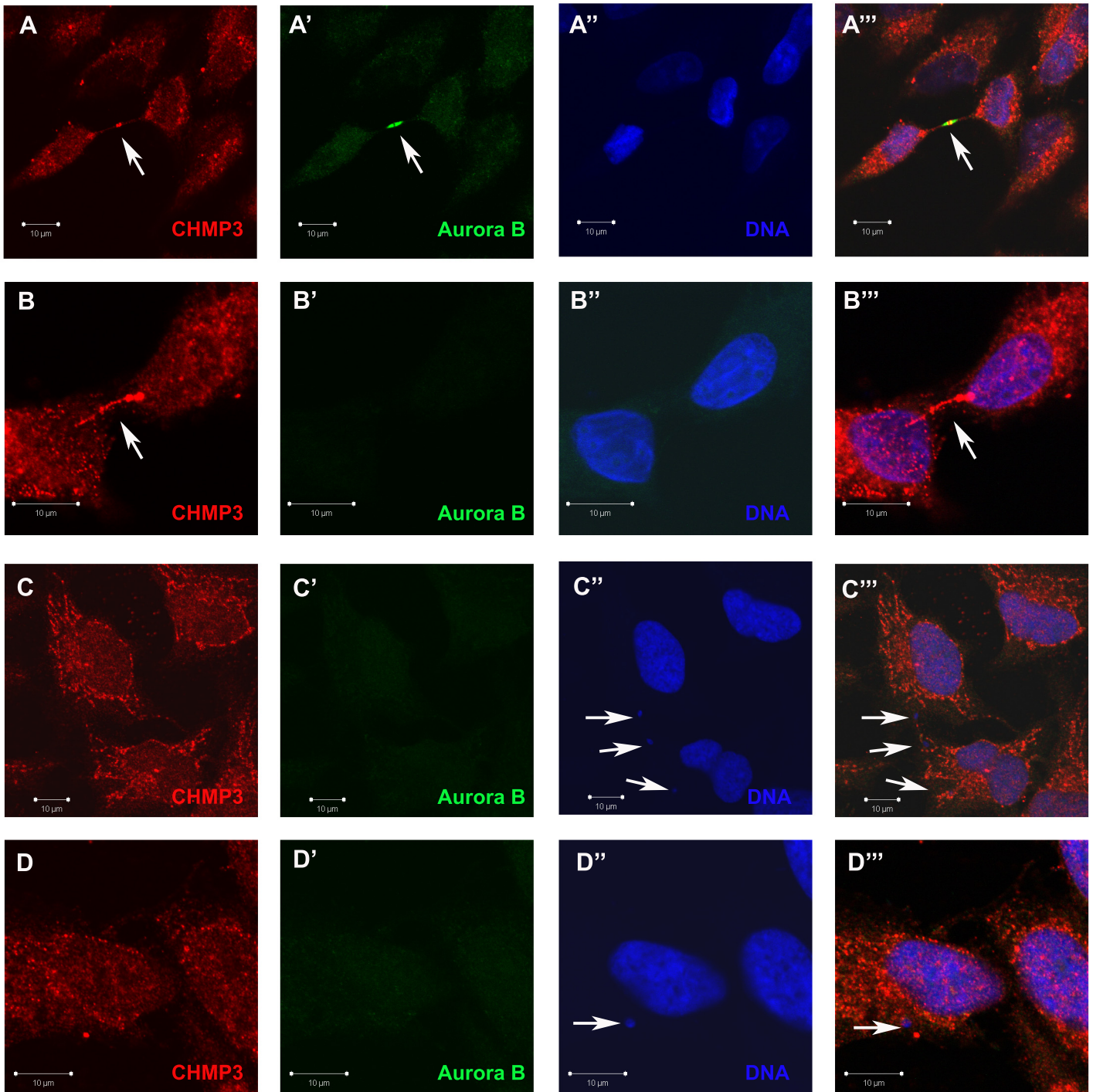

Suppl.fig.1 Sagona et al., 2011

**A**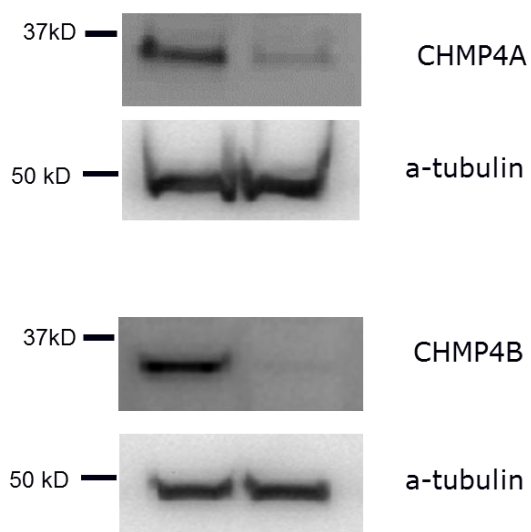**B**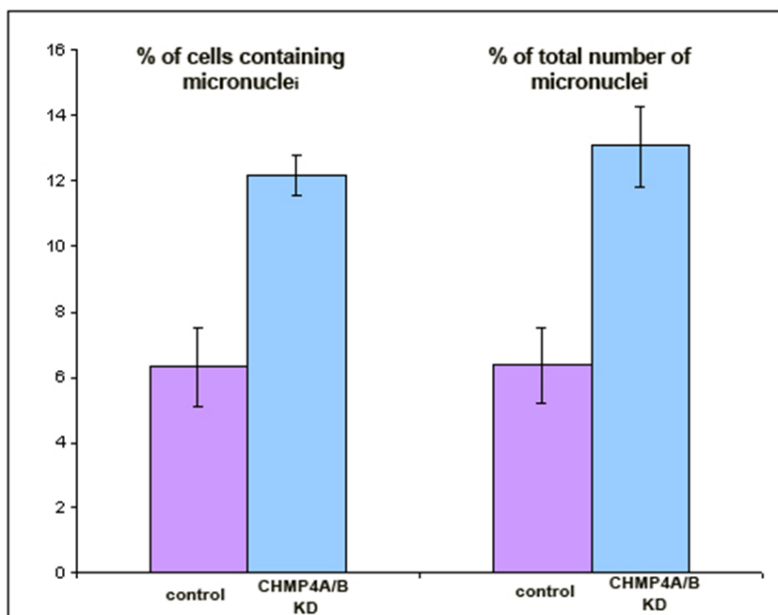**C**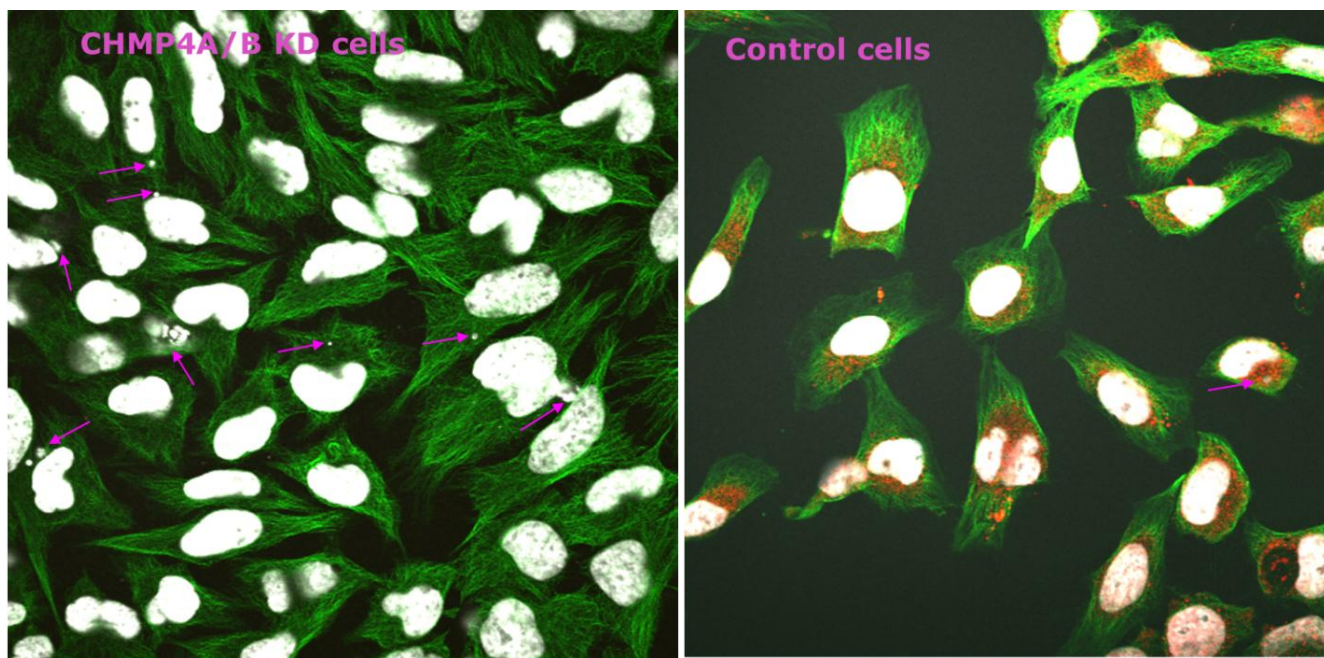

a-tubulin Hoechst CHMP4B

Supplement: Supplementary file 1 — Supplementary Figure 1: CHMP3 localizes at the midbody and intercellular bridge but does not colocalize with micronuclei. Supplementary Figure 2: Depletion of CHMP4A/B results in increased number of micronuclei. [file 974393.f1.zip › Suppl Materials.pdf]
